# Dopamine replacement therapy modulates DDC, prolactin and AOC3 levels in Parkinson’s disease

**DOI:** 10.1038/s41531-026-01435-3

**Published:** 2026-07-04

**Authors:** Ludo van Hillegondsberg, Shahzad Ahmad, Tanja Zerenner, Michael Lawton, Karolien Groenewald, Micah Fletcher, Ayan Ianniello, Yoav Ben-Shlomo, Richard Wade-Martins, Paolo Piazza, Avigail Taylor, Alexander G. Thompson, Michele T. Hu

**Affiliations:** 1 Nuffield Department of Clinical Neurosciences, University of Oxford, Oxford, UK; 2 Oxford-GSK Institute of Molecular and Computational Medicine, University of Oxford, Oxford, UK; 3 Population Health Sciences, University of Bristol, Bristol, UK; 4 Department of Physiology, Anatomy & Genetics, University of Oxford, Oxford, UK; 5 Centre for Human Genetics, University of Oxford, Oxford, UK

**Keywords:** Biomarkers, Diseases, Medical research, Neurology, Neuroscience

## Abstract

Dopa decarboxylase (DDC) is a candidate biomarker in Parkinson’s disease (PD), but its levels are influenced by dopamine replacement therapy (DRT), complicating interpretation. We used high-throughput proteomics to analyze serum and CSF samples from PD patients, individuals with idiopathic REM-sleep behaviour disorder, and controls to assess DRT effects. In serum, DDC increased and prolactin and AOC3 decreased with higher levodopa equivalent daily doses, showing drug class-specific associations. Longitudinally, serum DDC and prolactin levels changed with DRT, while AOC3 remained stable. In CSF, DDC was elevated regardless of treatment, while prolactin was reduced only in treated PD. These results indicate that serum changes in DDC, prolactin, and AOC3 primarily reflect treatment exposure rather than disease biology. Our findings highlight the need to account for medication effects in proteomic studies, identify AOC3 as a novel DRT-sensitive protein, and support the potential of treatment-responsive proteins as pharmacodynamic markers in PD.

## Introduction

Parkinson’s disease (PD) is a progressive neurodegenerative disorder characterized by the selective loss of dopaminergic neurons in the substantia nigra and the accumulation of alpha-synuclein protein aggregates, known as Lewy bodies, within neurons^1^. As the second most common neurodegenerative condition after Alzheimer’s disease, PD affects approximately 1% of people over 60 worldwide, with its motor, non-motor and cognitive symptoms imposing a significant burdens on patients, caregivers, and healthcare systems^2,3^. To elucidate the pathological mechanisms that underpin PD, recent years have witnessed a steady rise in the application of high-throughput proteomics in the PD field, leading to the discovery of numerous candidate biomarkers^4–7^. Among these, dopa decarboxylase (DDC), also known as aromatic L-amino acid decarboxylase (AADC), has emerged as a protein of particular interest. DDC has been shown to be elevated in the cerebrospinal fluid (CSF) of prodromal, treatment-naïve PD and treated PD in multiple cohorts and is positively correlated with clinical severity scores^8–10^. However, while DDC is also elevated in the plasma of people with PD, this increase is not observed in prodromal or early, treatment-naïve stages^11^. Furthermore, its lack of association with clinical severity outcomes, coupled with evidence of paradoxical upregulation in the blood of patients receiving L-dopa and peripheral decarboxylase inhibitors (PDIs), suggests that elevated plasma DDC is likely a consequence of dopamine replacement therapy (DRT) rather than a reflection of PD’s pathological mechanisms^12,13^. This case highlights the influence of drug intake on blood proteomic profiles, a largely underexplored area in patient populations. Understanding the protein-medication relationship will help to identify the off target effects of medications and elucidate the confounding role of medication use in biomarker discovery.

In this study, we investigated how DDC and other proteins beyond DDC may be modulated by dopamine replacement therapy (DRT). Using a proximity extension assay (PEA) proteomics platform, we conducted high-throughput proteomic analysis of serum and CSF of individuals with idiopathic REM-sleep behaviour disorder (iRBD), PD, and healthy controls (HC). We then conducted cross-sectional and longitudinal analyses to assess the impact of drug intake on protein levels.

## Results

### Study population demographics

Demographic and clinical characteristics are summarised in Table 1. Serum proteomic profiling was performed in 1368 cases (PD = 894, iRBD = 256, HC = 218). Among the PD participants, 96% had been diagnosed within the preceding 3.5 years, and 122 were untreated at the point of enrolment. A single longitudinal sample, collected 3-4 years later, was available for 42% of PD participants (373), 34% of iRBD participants (88) and 20% (44) of healthy controls. Disease severity scores were similar in treated and untreated PD participants and an overview of dopaminergic medication types can be found in Supplementary Table 1. CSF proteomic analysis included 83 individuals (PD = 50, iRBD = 14, HC = 19). There were no longitudinal CSF samples available. PD participants tended to be older, and the majority of those with iRBD were male. 49 participants had partially matched serum and CSF samples (samples taken within 1 year of each other [IQR: Q1 = 0.04, Q3 = 0.62]). Disease severity scores were similar in treated and untreated PD participants, and an overview of dopaminergic medication types can be found in Supplementary Table 2. Of the nine individuals with iRBD that phenoconverted during the follow-up window of 3–4 years, seven developed PD and two dementia with Lewy bodies (DLB). Four individuals had a baseline sample in both the iRBD and PD arms of the study. Mean time to phenoconversion from iRBD diagnosis was 6.26 years.

**Table 1:** Study population demographics

Serum Samples					
	Healthy control	iRBD	Parkinson’s disease		*p*-value
			Untreated	On treatment	
	(*n* = 218)	(*n* = 256)	(*n* = 122)	(*n* = 772)	
Age (years)	65 ± 6	66 ± 9	67 ± 9	68 ± 10	<0.001
Sex (no. male, %)	113 (52%)	227 (89%)	87 (71%)	482 (62%)	<0.001
Time since diagnosis (years)	-	1.4 ± 1.8	0.8 ± 0.8	1.33 ± 1	<0.001
UPDRS II	-	3 ± 5	6 ± 5	9 ± 6	<0.001
UPDRS III	1± 2	5 ± 4	27 ± 11	26 ± 11	0.5
Hoehn & Yahr stage (no., %)					0.2
1	-	-	33 (28%)	171 (22%)	
2	-	-	80 (67%)	535 (69%)	
3	-	-	5 (4.2%)	64 (8.3%)	
4-5	-	-	1 (0.8%)^b^	0	
MoCA^†^	27 ± 3	25 ± 3	25 ± 3	25± 3	0.5
LEDD (mg/day)	-	-	-	324 ± 189	
No. with longitudinal sample	44	88	58	315	
Time between samples (years)	4.1 ± 2.3	3.2 ± 0.3	3.2 ± 0.2	3.3 ± 0.3	0.5
Phenoconverted at follow-up	-	9	-	-	

CSF Samples					
	Healthy control	iRBD	Parkinson’s disease		*p*-value
			Untreated	On treatment	
	(*n* = **19**)	(*n* = **14**)	(*n* = **9**)	(*n* = **41**)	
Age (years)	65 ± 8	67 ± 8	65 ± 5	65 ± 9	0.6
Sex (no. male, %)	9 (47%)	12 (86%)	6 (67%)	25 (61%)	0.2
Time since diagnosis (years)	-	1.4 ± 1.8	0.8 ± 0.8	1.33 ± 1	0.02
UPDRS II	-	3 ± 4	5 ± 2	8 ± 5	0.2
UPDRS III	2± 3	4 ± 4	23 ± 11	26 ± 13	0.7
Hoehn & Yahr stage (no., %)					0.5
1	-	-	3 (33%)	8 (20%)	
2	-	-	6 (67%)	27 (66%)	
3	-	-	0	6 (15%)	
4-5	-	-	0	0	
MoCA^a^	27 ± 2	28 ± 2	26 ± 3	26 ± 2	0.7
LEDD (mg/day)	-	-	-	358 ± 213	
No. matched to serum	8	6	4	31	
Phenoconverted at follow-up	-	0	-	-	

Continues variables are reported as mean ± standard deviation. Baseline scores of disease severity markers reported, with accompanying *p*-values calculated using only the two PD groups.

^a^Montreal Cognitive Assessment, adjusted for years of education.

^b^Participant wheelchair-bound due to paralysis unrelated to PD. Participant excluded from Chi-squared test.

### Proteins associated with dopamine replacement therapy

Of 5,416 serum proteins, only three were significantly associated with DRT at baseline: dopa decarboxylase (DDC) (*β* = 0.96, *p-adjust* = 1.88 × 10^−32^), prolactin (PRL) (*β* = −1.03, *p-adjust* = 5.4 × 10^−6^), and amine oxidase copper-containing 3 (AOC3) (*β* = −0.21, *p-adjust* = 4.6 × 10^−5^)(Supplementary Fig. 1). In CSF, of 2,884 proteins, none were significantly associated with DRT at baseline (Supplementary Fig. 2). Correlations of serum and CSF DDC, PRL and AOC3 levels are displayed in Supplementary Fig. 3.

### Dopa decarboxylase

**Fig. 1:**
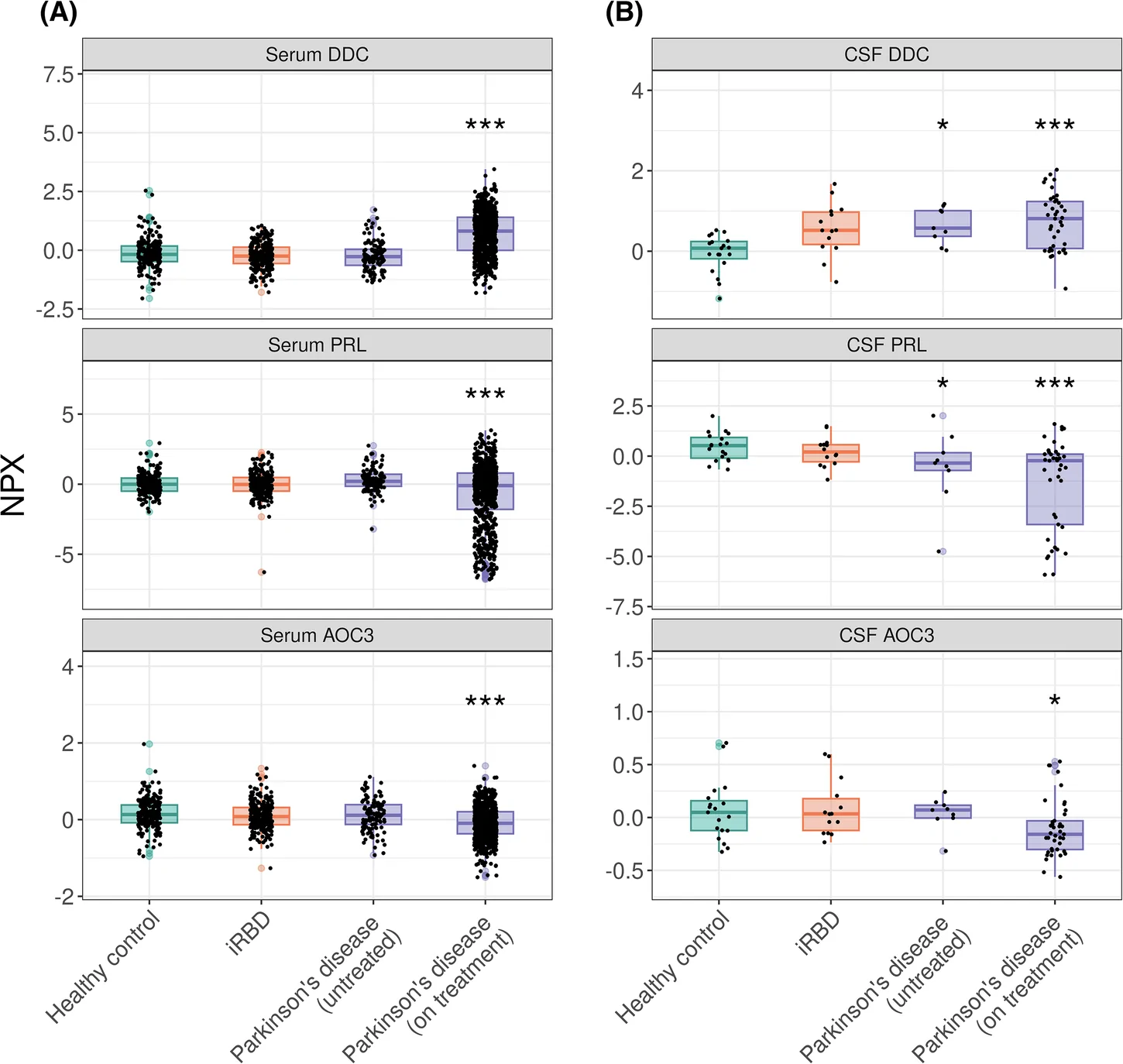
Protein levels by diagnostic group. **A** Raw serum protein NPX of all baseline samples.Simple linear model fitted. Covariates age, sex, BMI and technical variables. **B** Raw CSF protein NPX of all baseline samples. Simple linear model fitted. Covariates age, sex, BMI and technical variables. All comparisons are against the healthy control group. *p < 0.05,**p < 0.01***p < 0.001. All *p*-values FDR-corrected.

**Fig. 2:**
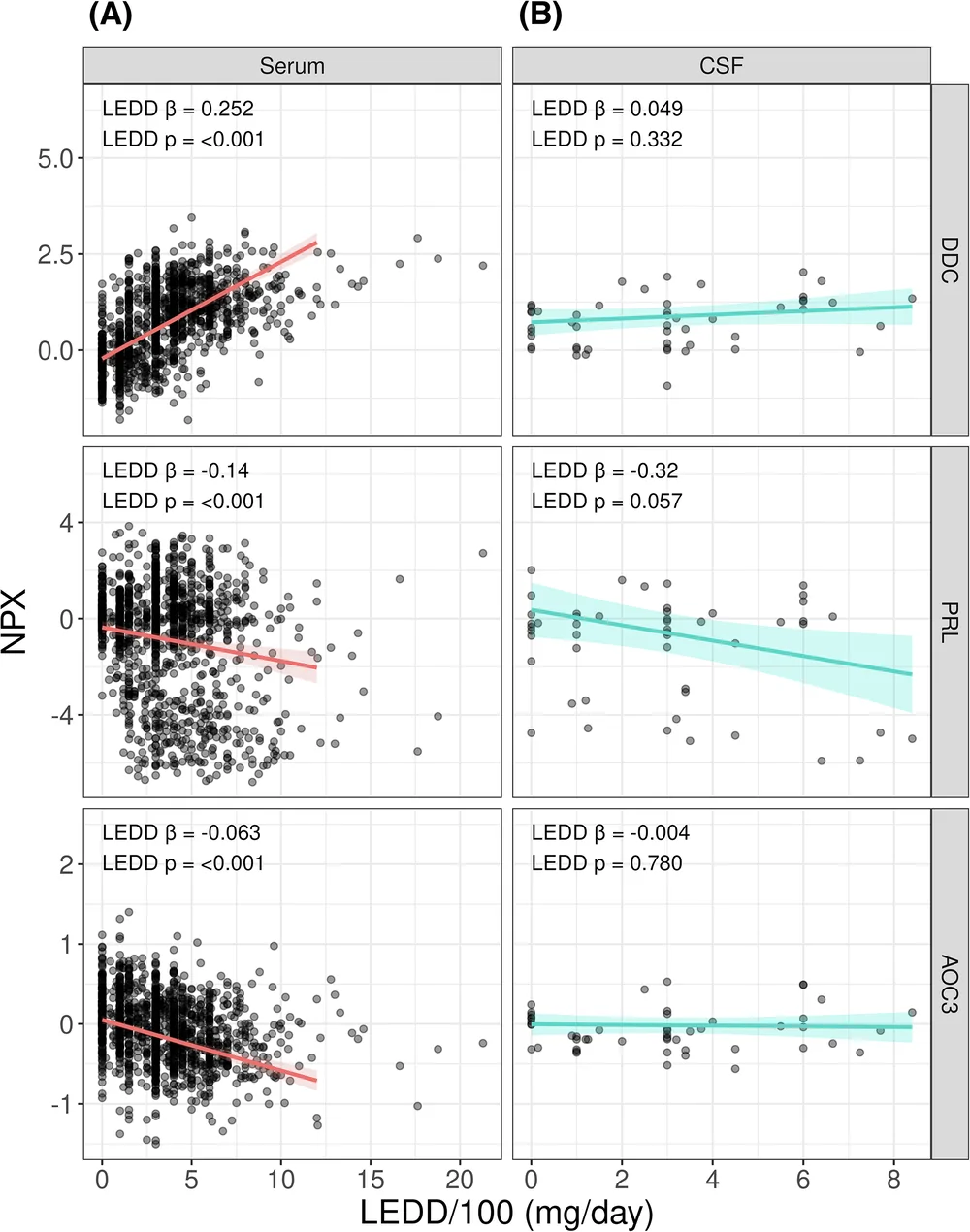
Linear associations between proteins and LEDD in serum and CSF. **A** Raw serum protein NPX of all PD samples (*n* = 1267). Linear mixed effects model fitted. Fixed effects: LEDD, disease duration, age, sex, BMI and technical variables. Random intercept: participant ID. **B** Raw CSF protein NPX of all baseline PD samples (*n* = 50). Simple linear model fitted. Covariates: age, sex, BMI and technical variables All *p*-values FDR-corrected.

**Fig. 3:**
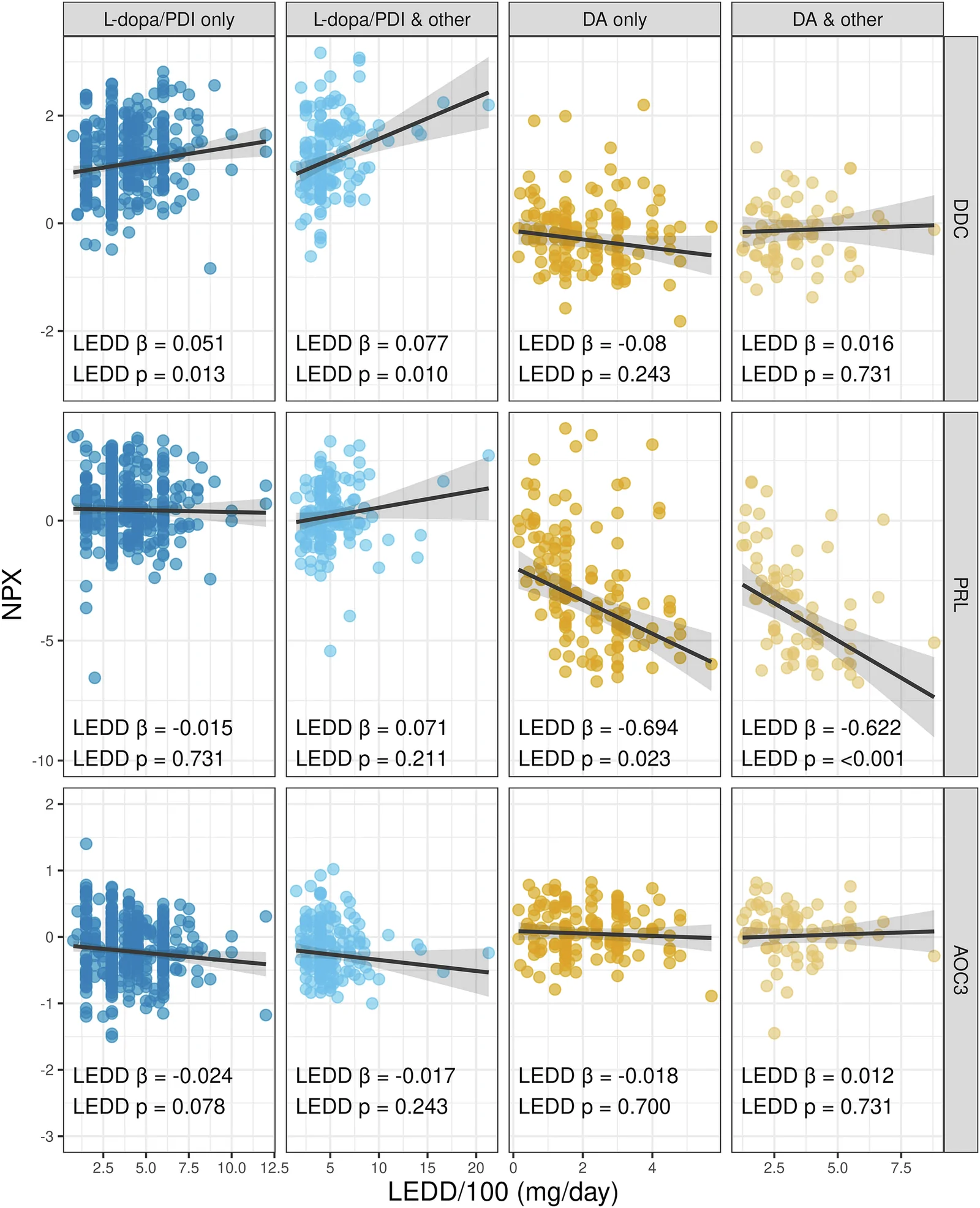
Linear associations between LEDD and serum protein levels stratified by drug class. Raw serum protein NPX of samples plotted. Coefficient of LEDD and related *p*-value displayed. Linear mixed effects model fitted. Fixed effects LEDD, disease duration, age, sex, BMI and technical variables Random intercept participant ID.L-dopa/PDI only: Participants only on a combination drug of L-dopa/PDI (*n* = 383) L-dopa/PDI & other: Participants on L-dopa/PDI and any other drug class, excluding dopamine agonists (*n* = 135) DA only: Participants only on a dopamine agonist (*n* = 132) DA & other: Participants on a dopamine agonist and any other drug class, excluding L-dopa/PDI (*n* = 64) Likelihood-ratio tests were used for the “DA & other” drug class models in which denominator degrees of freedom for fixed-effect t-tests could not be reliably estimated. All *p*-values FDR-corrected.

**Fig. 4:**
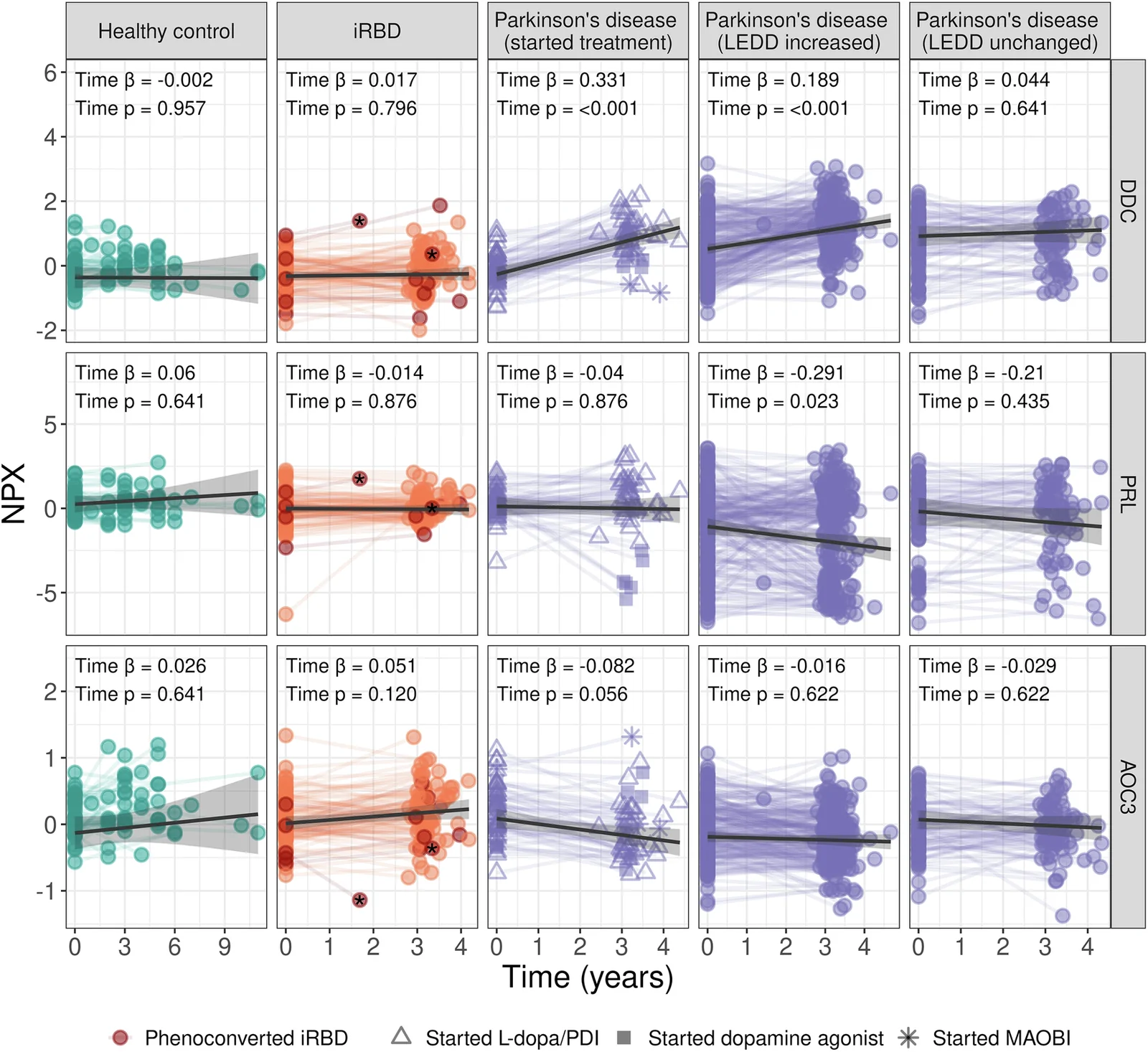
Change in serum protein levels over time stratified by change in LEDD. Raw serum protein NPX of samples plotted. Started treatment = 51, LEDD increased = 272, LEDD unchanged = 90.LEDD increased: > 100 mg increase in LEDD between visits. LEDD unchanged: −100 mg to 100 mg change in LEDD between visits. Coefficient of time between visits and related *p*-value displayed. Linear mixed effects model fitted. Fixed effects: time, baseline age, sex, BMI and technical variables. Random intercept: participant ID. All *p*-values FDR-corrected.* 2/9 iRBD phenoconverters initiated on L-dopa/PDI at time of repeat sampling. Started L-dopa/PDI = PD patient initiated on either L-dopa/PDI alone or L-dopa/PDI and any other DRT (excluding a dopamine agonist) at time of repeat sampling. Started dopamine agonist = PD patient initiated on either a dopamine agonist alone or a dopamine agonist and any other DRT (including L-dopa/PDI) at time of repeat sampling. MOABI = monoamine oxidase B inhibitor.

In serum, baseline DDC levels were significantly elevated in individuals with PD compared to healthy controls, but only in those on treatment (*β* = 0.79, *p-adjust* = 1.6 × 10^−28^) (Fig. 1). Serum DDC levels were positively associated with LEDD in PD (*β* = 0.19 per 100 mg, *p-adjust* = 9.8×10^−62^), an association that was specific to PD participants on L-dopa/PDI when data was grouped by drug class (Figs. 2 and 3). The association between serum DDC level and LEDD remained significant when adjusting for disease severity metrics (Table 2). In longitudinal serum samples, individuals with PD that started treatment or had an increase in their LEDD exhibited a rise in DDC levels at the subsequent visit (started treatment: *β* = 0.33, *p-adjust* = 7.5 × 10^−7^, LEDD increased: *β* = 0.19, *p-adjust* = 4.05 × 10^−7^), with the rise most evident in PD participants that started L-dopa/PDI or had L-dopa/PDI added to their drug regimen (Fig. 4 and Supplementary Fig. 5). CSF DDC levels were elevated in PD, regardless of treatment status (treated: *β* = 0.80, *p-adjust* = 0.0002, untreated: *β* = 0.68, *p-adjust* = 0.027). No significant association was observed between CSF DDC levels and LEDD; however, CSF DDC was associated with UPDRS II and Hoehn & Yahr stage (Supplementary Table 3). DDC levels were broadly unchanged in the iRBD group in both serum and CSF (Figs. 1–3), however the two phenoconverted individuals that started L-dopa/PDI saw a rise in their DDC levels (Fig. 4). iRBD patients that had phenoconverted at follow-up did not demonstrate higher levels of serum DDC at baseline (Supplementary Fig. 4).

### Prolactin

Baseline serum prolactin levels were significantly reduced in participants with PD, but only in those on treatment (*β* = −0.89, *p-adjust* = 4.12 × 10^−8^) (Fig. 1). Serum prolactin levels were negatively associated with LEDD in PD (*β* = −0.12 per 100 mg, *p-adjust* = 9.04 × 10^−6^), a relationship specific to PD participants on dopamine agonists when stratified by drug class (Figs. 2 and 3). The association between serum prolactin level and LEDD remained significant when adjusting for disease severity metrics (Table 2). In longitudinal serum samples, individuals with PD that had an increase in their LEDD exhibited a decrease in prolactin levels at the subsequent visit (*β* = −0.29, *p-adjust* = 0.023), with the decline most evident in PD participants that started a dopamine agonist or had a dopamine agonist added to their drug regimen (Fig. 4 and Supplementary Fig. 5). Prolactin levels in CSF were significantly lower in the treated PD group, and to a lesser degree, the untreated group (treated: *β* = −2.19, *p-adjust* = 0.0002, untreated: *β* = −1.67, *p-adjust* = 0.047). No significant association was observed between CSF prolactin levels and LEDD or any disease severity metrics (Supplementary Table 3). Prolactin levels were unchanged in the iRBD group in both serum and CSF (Figs. 1 - 3).

### AOC3

Baseline AOC3 levels were reduced only in the serum of participants with PD receiving treatment (*β* = −0.24, *p-adjust* = 4.36 × 10^−11^) (Fig. 1). Serum AOC3 levels were negatively associated with LEDD in PD (*β* = −0.04 per 100 mg, *p-adjust* = 6.24 × 10^−18^); however, when stratified by drug class, no association met the significance threshold (Figs. 2 and 3). The association between serum AOC3 level and LEDD remained significant when adjusting for disease severity metrics (Table 2). In longitudinal serum samples, no group showed a significant change in AOC3 at follow-up, however in the subgroup analysis where PD participants had L-dopa/PDI added to their drug regimen, there was a significant decline in AOC3 levels (*β* = −0.076, *p-adjust* = 0.001) (Fig. 4 and Supplementary Fig. 5). In CSF, AOC3 levels were reduced only in PD individuals on treatment (*β* = −0.16, *p-adjust* = 0.0471). No significant association was observed between CSF AOC3 levels and LEDD or any disease severity metrics (Supplementary Table 3). AOC3 levels were unchanged in the iRBD group in both serum and CSF (Figs. 1–3).

**Table 2:** LEDD and clinical variables

Serum DDC			
Model	Variable	Coefficient (*β*)	*p*-value^a^
LEDD	LEDD	0.192	3.25e^-62^
LEDD + UPDRS II^b^	LEDD	0.193	1.64e^-59^
LEDD + UPDRS III^c^	LEDD	0.193	9.00e^-61^
LEDD + H&Y^d^	LEDD	0.192	1.11e^-61^
LEDD + MoCA^e^			
	LEDD	0.191	5.53e^-60^
**Serum PRL**			
**Model**	**Variable**	**Coefficient** **(*β*)**	***p*-value**^**a**^
LEDD	LEDD	−0.117	9.04e^-17^
LEDD + UPDRS II^b^	LEDD	−0.121	6.55e^-6^
LEDD + UPDRS III^c^	LEDD	−0.115	1.57e^-5^
LEDD + H&Y^d^	LEDD	−0.117	1.09e^-5^
LEDD + MoCA^e^	LEDD	–0.114	1.75e^-5^
**Serum AOC3**			
**Model**	**Variable**	**Coefficient** **(*β*)**	***p*-value**^**a**^
LEDD	LEDD	−0.044	4.16e^-18^
LEDD + UPDRS II^b^	LEDD	−0.043	1.17e^-17^
LEDD + UPDRS III^c^	LEDD	−0.044	5.98e^-18^
LEDD + H&Y^d^	LEDD	−0.044	2.42e^-18^
LEDD + MoCA^e^	LEDD	−0.044	4.12e^-18^

Associations of proteins with disease severity markers and LEDD were analysed by linear-mixed effects models. All models include disease duration, age, sex, BMI technical variables as fixed effects and a random intercept for participant ID. LEDD is shown scaled as LEDD/100 (mg/day).

*H&Y* Hoehn & Yahr stage.

^a^nominal *p*-value.

^b^*n* individuals = 886, *n* samples = 1236.

^c^*n* individuals = 880, *n* samples = 1238.

^d^*n* individuals = 890, *n* samples = 1260.

^e^*n* individuals = 887, *n* samples = 1245.

## Discussion

In this study, we conducted proteomic analysis of a large longitudinal cohort, examining both serum and CSF samples from individuals with PD, iRBD, and healthy controls. The key findings included alterations in the serum levels of DDC, prolactin, and AOC3 in individuals with PD on dopaminergic treatment, with more subtle changes observed in the CSF. Notably, the relationship between these three proteins, LEDD and dopaminergic drug classes varied both by protein type and biofluid, suggesting distinct mechanisms that underlie the alterations observed in the systemic and central nervous systems.

Serum and CSF DDC levels were elevated in people with PD versus controls. However, unlike in CSF, increased serum DDC was only observed in patients on treatment, with levels rising in a dose-dependent manner, particularly in those on L-dopa/PDI. Further added to this, serum DDC levels only showed significant change longitudinally in those that started DRT (of which 39/51 started on L-dopa/PDI) or saw an increase in their LEDD due to the addition of L-dopa/PDI to their drug regimen. These associations implicate L-dopa/PDI, not disease-related biology, as the primary driver of elevated serum DDC in PD. In contrast, analysis of our limited CSF samples revealed elevated DDC levels in both untreated and treated PD patients, associations between CSF DDC levels and disease severity metrics, and, although not statistically significant, higher CSF DDC levels in individuals with iRBD compared with controls. Collectively, these findings suggest that CSF DDC is a genuine PD biomarker, consistent with prior reports, and serves as a promising diagnostic marker of both prodromal and manifest PD^8–12,14^. Our findings thus strengthen the previously proposed hypothesis that elevated CSF DDC levels reflect PD disease pathophysiology, while elevated serum DDC levels indicate DRT–and specifically L-dopa/PDI–exposure^13,15^.

Prolactin levels in both serum and CSF were reduced only in PD patients. In serum, this reduction was observed only in those receiving DRT, and in a dose-dependent manner. Notably, dopamine agonists, rather than L-dopa/PDI medications, appeared to be the primary drivers of the correlation between LEDD and prolactin. Furthermore, serum prolactin levels decreased further at the subsequent visit only in patients who started a dopamine agonist, reinforcing the notion that DRT modulates prolactin levels. This phenomenon aligns with established physiology: dopamine inhibits prolactin release from the pituitary via the tuberoinfundibular pathway, and dopamine agonists would thus enhance this suppression^15^. Previous studies on older dopamine agonists, such as bromocriptine, pergolide, and lergotrile, have demonstrated this inhibitory effect on prolactin in PD, and more recently ropinirole has even been proposed a treatment for hyperprolactinaemia^16–19^. In untreated PD patients, while serum prolactin levels have yielded some conflicting results across previous studies, the majority would indicate prolactin levels are normal^20,21^, which is also reflected in our results. In line with what we would expect, CSF prolactin levels were clearly lower in the treated PD group (*p-adjust* = 0.0002); however, prolactin levels were surprisingly also lower in the treatment-naïve group (*p-adjust* = 0.047). Given that prolactin levels in untreated PD patients were normal in the much larger serum experiment and serum prolactin is known to closely correlate with CSF prolactin^22,23^, this finding may indicate a false positive result. With regard to the possible effects of PDIs, previous evidence from small case-control studies would suggest that serum prolactin levels may be reduced by these medications^24,25^. While one may expect some degree of prolactin suppression when CSF dopamine levels are elevated by any dopaminergic therapy, our results suggest that only in patients on dopamine agonists is a systematic reduction in prolactin levels demonstrable, which may be related to the longer half-life and receptor binding time of commonly used dopamine agonists such as ropinirole. Taken together, we postulate that prolactin levels are likely normal in PD, are unlikely to reflect disease biology, and are more likely modulated by DRT in a dose-dependent manner, with dopamine agonists exerting the strongest suppressive effect. Given the close correlation between serum and CSF prolactin, these therapy-related changes are likely mirrored in CSF to a similar extent. As neither dopamine agonists nor L-dopa/PDIs are known to have disease-modifying effects, lowering of PRL is unlikely to influence disease progression^26,27^.

The AOC3 levels were reduced in the serum and CSF of PD individuals on DRT only. AOC3, previously known as copper-dependent semicarbazide-sensitive amine oxidase (SSAO) or vascular adhesion protein-1 (VAP-1), is a membrane-bound adhesion protein expressed in hepatic endothelium and smooth muscle^28^. While its exact function is unclear, it is associated with vascular diseases, diabetes mellitus, and pulmonary, renal and liver conditions, where it likely, at least in part, acts as a pro-inflammatory mediator^28–32^. To our knowledge, there are only two studies that have examined AOC3 in relation to PD, which reported sustained inhibition of serum AOC3 activity following the administration of PDIs, and irreversible binding of benserazide to AOC3^33,34^. While enzymatic activity and protein levels are related but not equivalent, this previously described link between PDIs and serum AOC3 activity provides supportive context for our findings. Although the “L-dopa/PDI only” subset (Fig. 3) was not significantly associated with AOC3 levels, overall LEDD was negatively associated with AOC3, with most samples (822/1267, 65%) from participants on L-dopa/PDI. Furthermore, in the longitudinal analysis, the decline in AOC3 levels was most evident in the PD group initiating treatment, where 39/51 (76%) of participants began L-dopa/PDI and those that had L-dopa/PDI added to their drug regimen. Collectively, these results suggest that PDIs are likely the driver of the negative association between dopaminergic replacement therapy (DRT) and serum AOC3 levels. Notably, no significant change in AOC3 levels was observed in participants already on treatment, suggesting that the AOC3-DRT interaction may not be strongly dose-dependent, and that a plateau effect occurs after initial exposure. The mechanism underlying AOC3 downregulation remains unclear; however, we hypothesize that the previously reported irreversible binding of PDIs to AOC3 may trigger degradation of the protein, resulting in a modest but detectable reduction in overall AOC3 levels. On the question of whether lower serum AOC3 affects disease progression, this is unlikely, as long-term L-dopa/PDI use does not affect disease progression^26^. Downregulation of AOC3 was also observed in CSF (*p-adjust* = 0.0471). Given that PDIs are not expected to cross the blood-brain barrier to a significant extent, this finding suggests that the alteration in CSF AOC3 levels is either related to other DRT drug classes, PD disease biology, or represents a false positive result. It has been proposed that the pro-inflammatory actions of AOC3 contribute to disease progression in iRBD, a hypothesis that is currently being evaluated in an ongoing clinical trial^35^. However, larger CSF proteomic studies in PD have not identified AOC3 as a significant biomarker, and therefore, we interpret this result with caution. In summary, we hypothesise that perturbations in AOC3 levels in treated PD are driven by PDIs, possibly via irreversible binding and subsequent protein downregulation. The reduction in serum appears to occur early after treatment initiation, is not strongly dose-dependent, and is unlikely to influence disease progression, while the CSF finding should be interpreted cautiously.

While our findings demonstrate that modulation of DDC, prolactin, and AOC3 is associated with LEDD, our study does not establish direct causality. A critical consideration is whether LEDD serves as a surrogate for disease severity, given that individuals with more severe disease are typically on a higher LEDD. To address this, we incorporated disease duration–along with either UPDRS II, UPDRS III, H&Y stage, or MoCA scores–into our linear mixed-effects models (Table 2). The strong associations with LEDD persisted, with coefficients remaining largely unchanged.

It is prudent to note that this study is subject to several limitations that warrant consideration. The first pertains to the CSF sample size. Although the findings align broadly with those of previous studies, lending a degree of confidence to the results, the CSF sample size in this study is notably small. Consequently, any conclusions drawn from the data should be interpreted with caution. The second limitation concerns the challenge of disentangling the relationship between LEDD and disease severity. LEDD exhibits a natural correlation with disease severity, and the rarity of clinical or research scenarios in which individuals with PD abstain from DRT for an extended duration precludes the feasibility of examining a “DRT-free” cohort, hindering efforts to isolate the independent effects of LEDD. Compounding this issue, the current instruments employed to assess disease severity—such as the UPDRS and H&Y—are imperfect. As a result, incorporating these metrics into analytical models to adjust for disease severity is not robust. A further limitation of this study stems from the use of NPX units to measure protein levels, a metric that provides relative rather than absolute quantification. As a result, observed variations in protein expression are intrinsically tied to the specific experimental conditions of this study, limiting their broader applicability. In the absence of bridging samples, this approach poses significant challenges for directly comparing our findings with those of other studies.

To conclude, our findings indicate that dopamine replacement therapy modulates DDC, PRL, and AOC3 levels in PD. The distinct patterns of these alterations in serum and CSF are driven by specific drug classes and align with known effects of peripheral decarboxylase inhibitors and dopamine agonists. The drug-related change in serum AOC3 levels, demonstrated here in a large cohort, represents a unique and significant finding and highlights the therapeutic potential of PDIs as an inhibitor of AOC3. More broadly, our results emphasize the importance of understanding the impact of dopamine replacement therapy on the PD proteome, and its potential to obscure the separation of treatment effects from disease biology in proteomic studies. Disentangling these influences is not only essential for identifying reliable biomarkers but may also advance the clinician’s ability to monitor those on treatment.

## Methods

### Study population

This study utilized the Discovery Cohort from the Oxford Parkinson’s Disease Centre, a community-based longitudinal cohort of approximately 1900 participants recruited over 14 years, primarily from the Thames Valley region in the United Kingdom. This cohort consists of three main arms: people with PD, individuals with iRBD, and healthy controls. Participants with PD were diagnosed by an NHS neurology consultant prior to enrolment, with their diagnosis confirmed at enrolment by a neurology consultant or fellow using the UK Brain Bank Criteria in a structured interview and examination^36^. Participants were reviewed every 12–18 months, and those who developed atypical parkinsonian syndromes were withdrawn. Individuals with iRBD had their diagnosis confirmed via polysomnography (PSG) before enrolment, in accordance with the International Classification of Sleep Disorders criteria^37^. iRBD is widely recognized as a prodromal manifestation of PD, with the most extensive multi-cohort study conducted to date finding that 73.5% of individuals with iRBD developed a synucleinopathy within 12 years of follow-up^38–40^. Healthy controls exhibited no evidence of neurodegenerative disease at baseline neurological review, had no family history of PD, underwent routine monitoring every 12–18 months and were withdrawn if a neurodegenerative disease developed. Levodopa equivalent daily dose (LEDD) was calculated for each participant using established conversion formulas^41,42^. Full details of the clinical protocol have been published elsewhere^43^. The cohort study protocol complied with the Declaration of Helsinki, received approval from the NHS Health Research Authority’s local ethics committee (South Central—Oxford A, REC reference number 16/SC/0108), and all participants provided written informed consent.

### Proteomic analysis and quality control

Proteomic analysis was performed using proximity extension assay (PEA) technology (Olink Proteomics, Uppsala, Sweden). This method enables the detection of proteins using specific antibodies, each conjugated to a unique oligonucleotide sequence corresponding to a target protein in the panel. When these antibodies bind to their respective proteins, the paired oligonucleotides hybridize, allowing amplification via polymerase chain reaction. These amplicons are then quantified by next-generation sequencing and converted into normalized protein expression (NPX) values, reported on a log2-scale, reflecting the relative abundance of each protein in the sample^44^. Serum samples were analysed with the Olink Explore HT platform, while CSF samples were analysed with the Olink Explore 3072 platform. The analysis was conducted by an independent team at the Centre for Human Genetics, University of Oxford, who were blinded to all clinical data. Sample plate layouts for serum and CSF were randomized. In the larger serum experiment, the layout of each plate was programmatically generated using the concept of negative spatial autocorrelation^45^. Raw data acquisition and quality control (QC) followed the manufacturer’s protocol^46,47^. Additional QC steps were employed to identify sample outliers and calculate assay performance metrics. In the serum experiment, of 5416 assays, 1810 (33.42%) had <30% of results below the lower limit of detection (LOD) with a mean interplate coefficient of variance (CV) of 13.70%. In the CSF experiment, of 2884 assays, 1532 (53.12%) had <30% of results below the LOD with a mean interplate CV of 18.68%. A summary of the QC steps, sensitivity analyses and other relevant metrics can be found in the supplementary material (Supplementary Fig. 6-11, Supplementary Table 4).

### Statistical analysis

Demographic characteristics of the study population were analysed to assess baseline differences. Distributions of continuous variables across groups were compared using the Kruskal-Wallis test and binary variables were evaluated with Pearson’s Chi-squared test. To investigate the association between protein levels and therapy status at baseline, we performed linear regression analyses with protein level as the dependent variable and DRT status (regardless of diagnostic group) as the primary independent variable. All models were adjusted for diagnosis, age, sex, body mass index (BMI), and technical factors (sample age and the number of sample freeze-thaw cycles). Of the proteins associated with DRT, protein levels in each diagnostic group were compared against the healthy control group using a linear regression model, with age, sex, BMI and the aforementioned technical variables as covariates. The relationship between drug dosage, measured in levodopa equivalent daily dose (LEDD), and protein level in CSF was determined using a linear regression model, with age, sex, BMI and technical factors as covariates. In serum, the relationship between protein levels and drug dosage was assessed using both baseline and longitudinal samples, and so a linear mixed effects model was employed. Fixed effects comprised of drug dose, disease duration, age, sex, BMI, and technical variables, with participant ID as a random intercept to account for intra-individual correlation. Markers of disease severity were added as fixed effects to determine the associations between protein levels and LEDD when accounting for disease severity. To evaluate longitudinal changes in protein levels, a separate linear mixed effects model was fitted: time (interval between measurements), baseline age, sex, BMI, and technical variables were incorporated as fixed effects, with participant ID specified as a random intercept to address repeated measures within individuals. Where applicable, the *p*-value was adjusted using the Benjamini-Hochberg false-discovery rate (FDR) correction method and considered significant at < 0.05. All statistical analyses were performed using R (version 4.4.2). Linear mixed effects models were implemented with the *nlme* package (version 3.1.166).

## Supplementary information

npj_Supplement_REVISED.

## Data Availability

The datasets generated and/or analysed during the current study are not publicly available due to participant confidentiality, consent limitations and the proprietary nature of the data, but are available from the corresponding author on reasonable request.

## Code availability

The underlying code for this study is available on GitHub and can be accessed via this link: https://github.com/LudoVH/Public_code/blob/main/Olink_drug_effect.
