# Supplementary material for: Dopamine replacement therapy modulates DDC, prolactin and AOC3 levels in Parkinson’s disease
Source: NPJ Parkinsons Dis. 2026 Jul 4;12:263. doi: 10.1038/s41531-026-01435-3 (PMC13714277; doi:10.1038/s41531-026-01435-3)
Supplement: Supplementary file 1 [file 41531_2026_1435_MOESM1_ESM.pdf]

SUPPLEMENTARY MATERIAL

Supplementary Figures & Tables

Supplementary Figure 1. Proteins associated with DRT in serum

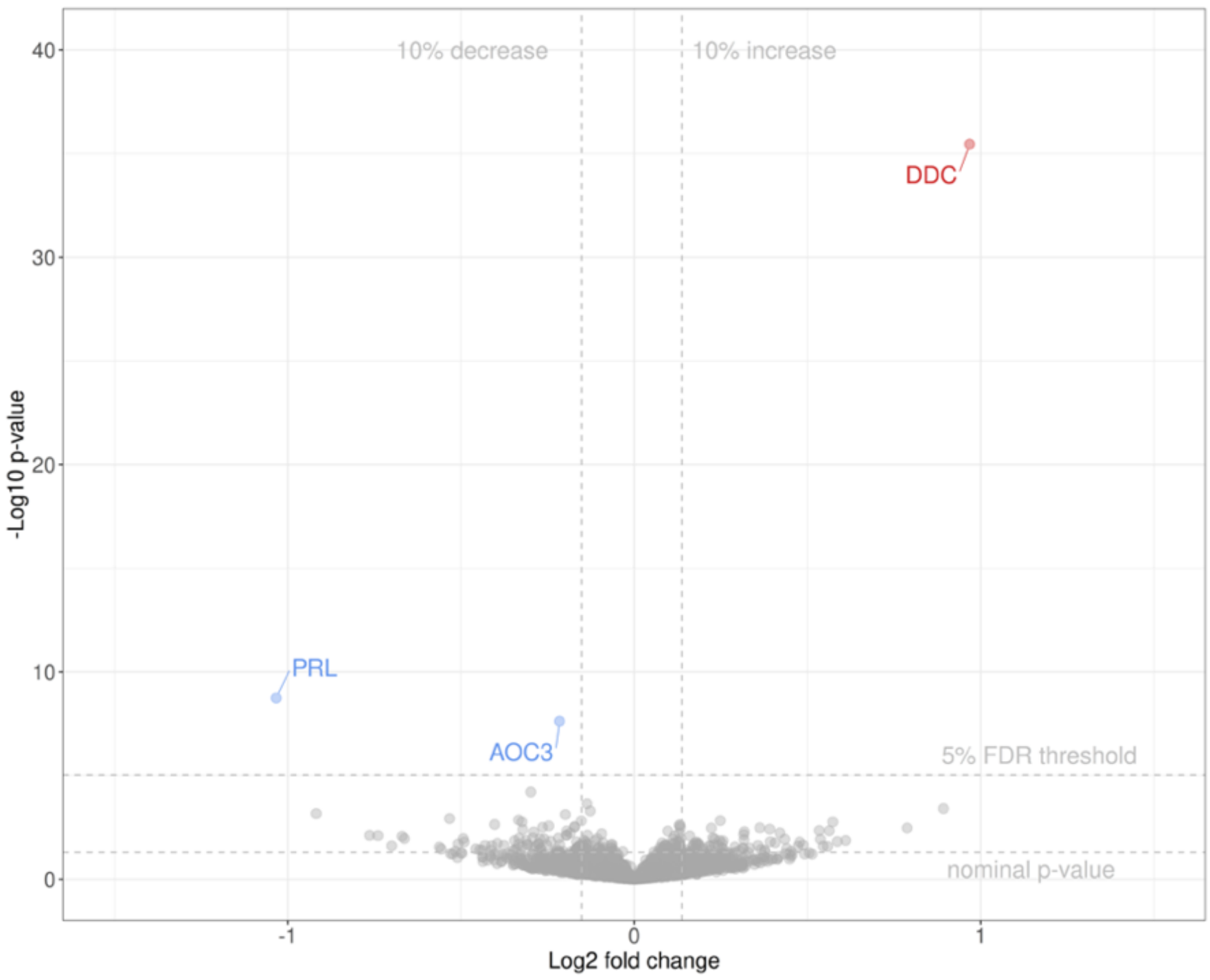

Simple linear model fitted to identify proteins associated with DRT at the baseline visit (covariates: diagnosis, sex, age, BMI, technical variables). DRT = Dopamine replacement therapy. DDC = dopa decarboxylase. PRL = prolactin. AOC3 = amine oxidase copper-containing 3

Supplementary Figure 2. Proteins associated with dopamine replacement therapy in CSF

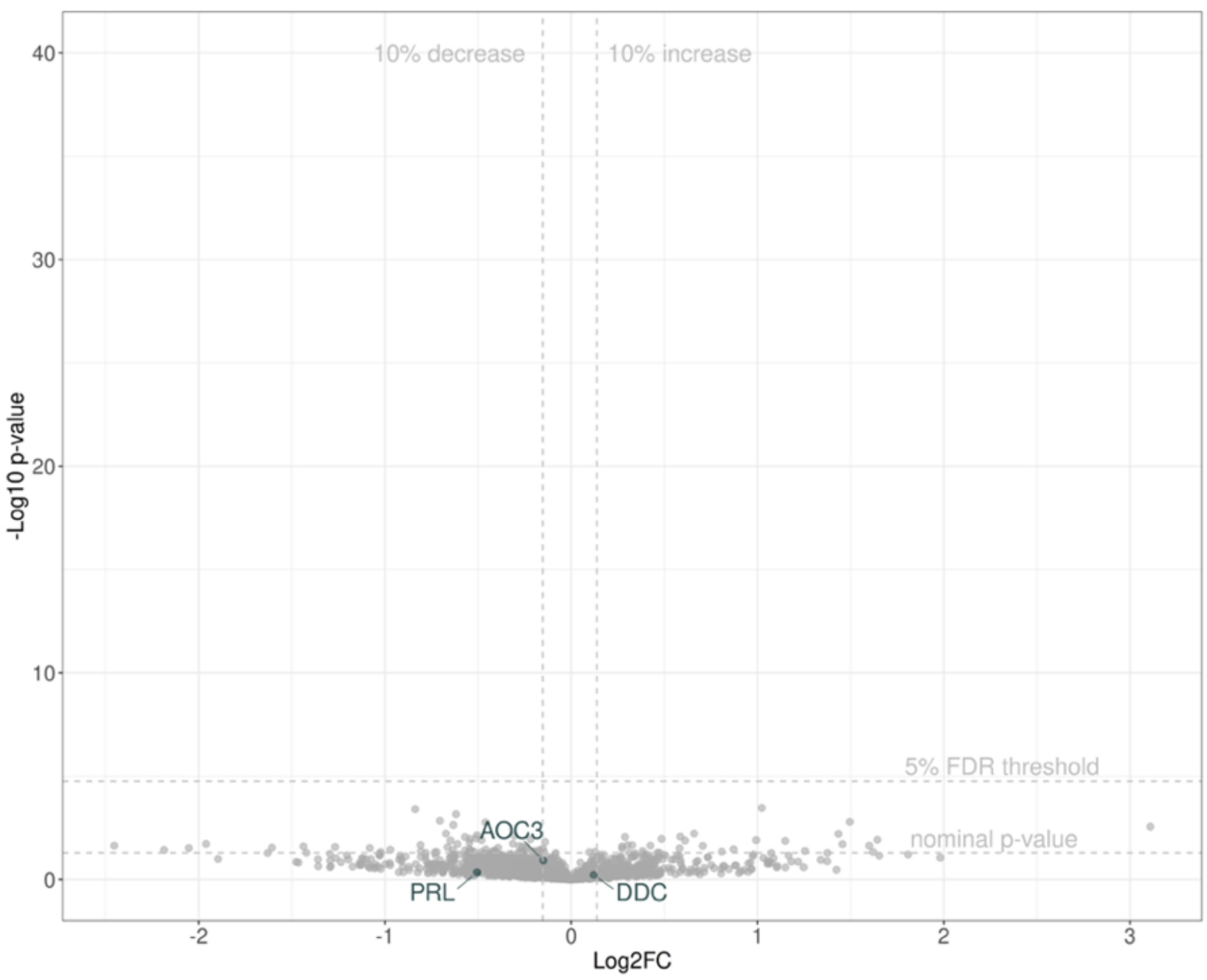

Simple linear model fitted to identify proteins associated with DRT at the baseline visit (covariates: diagnosis, sex, age, BMI, technical variables). DRT = Dopamine replacement therapy. DDC = dopa decarboxylase. PRL = prolactin. AOC3 = amine oxidase copper-containing 3

Supplementary Table 1. Serum proteomic data: Overview of dopaminergic medication classes

PD participants (baseline only)

Drug Class	Count
Levodopa/PDI	822
Dopamine agonist	411
MAOB inhibitor	368
COMT inhibitor	36
Amantadine	17

PD participants (baseline + follow-up)

Drug Class	Count
Levodopa/PDI	511
Dopamine agonist	263
MAOB inhibitor	251
COMT inhibitor	31
Amantadine	14

Supplementary Table 2. CSF proteomic data: Overview of dopaminergic medication classes

PD participants (baseline)

Drug Class	Count
Levodopa/PDI	27
Dopamine agonist	14
MAOB inhibitor	12
COMT inhibitor	1
Amantadine	0

Supplementary Figure 3. Correlation between serum and CSF NPX

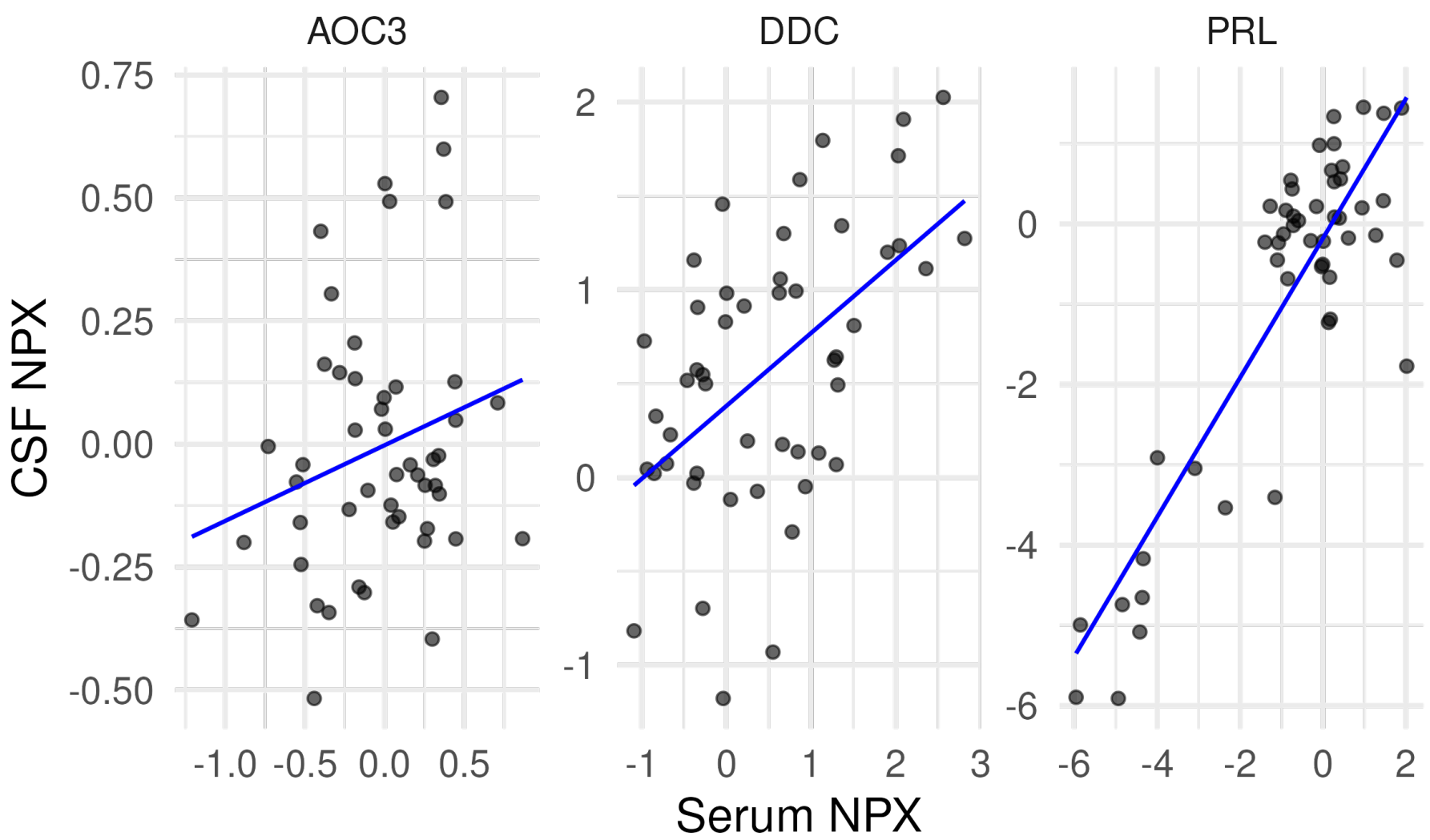

Total of 49 participants (PD = 35, iRBD = 6, HC = 8) with CSF and serum taken within 1 year.

Supplementary Figure 4. Serum protein level by diagnostic group

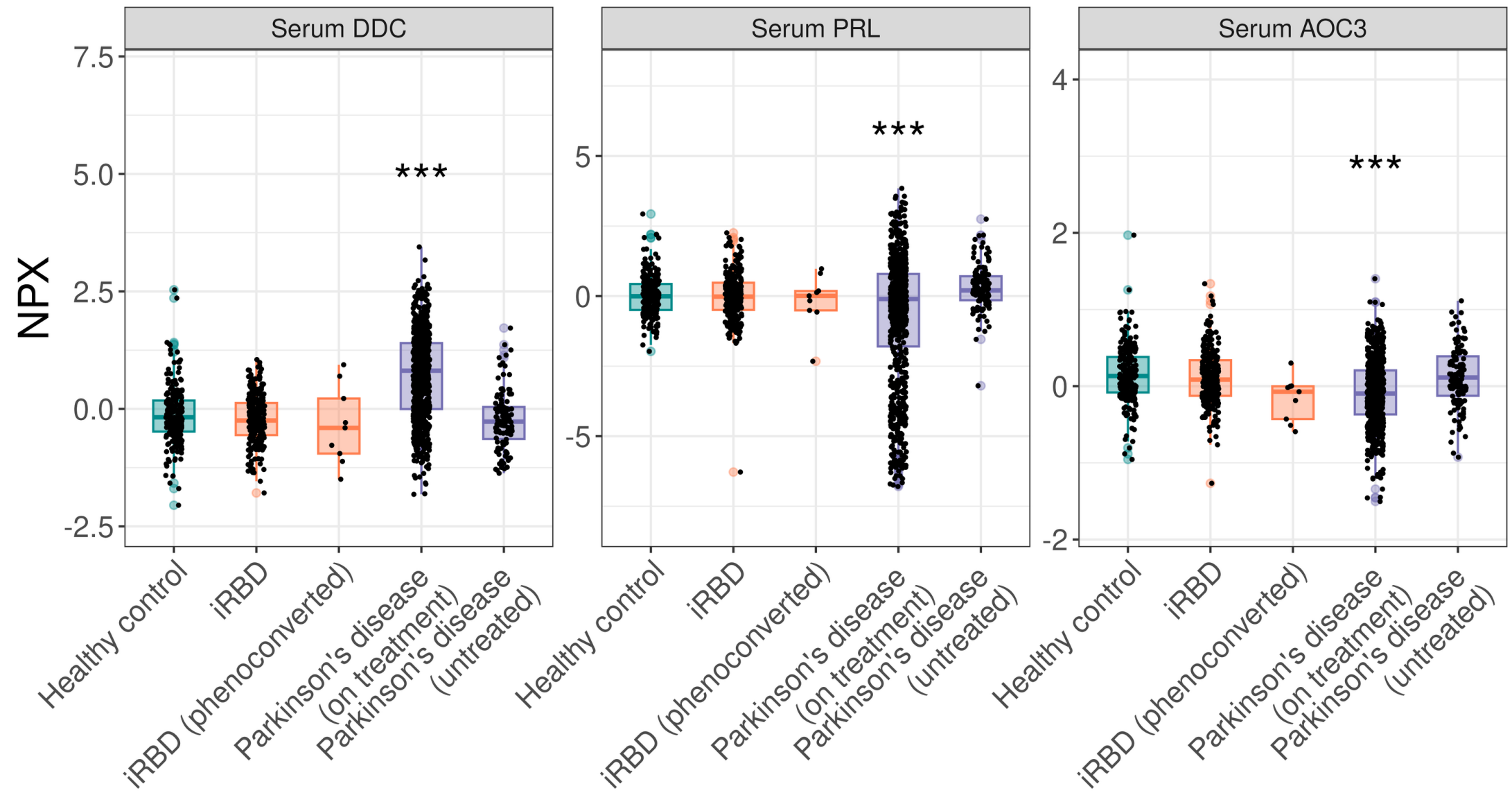

*Raw serum protein NPX of all baseline samples. Simple linear model fitted. Covariates age, sex, BMI and technical variables. All comparisons are against the healthy control group. * p < 0.05, ** p <0.01 *** p < 0.001. All p-values FDR-corrected.*

Supplementary Figure 5. Change in serum protein levels over time in PD patients that saw an increase in LEDD, stratified by drug class and subsequent change

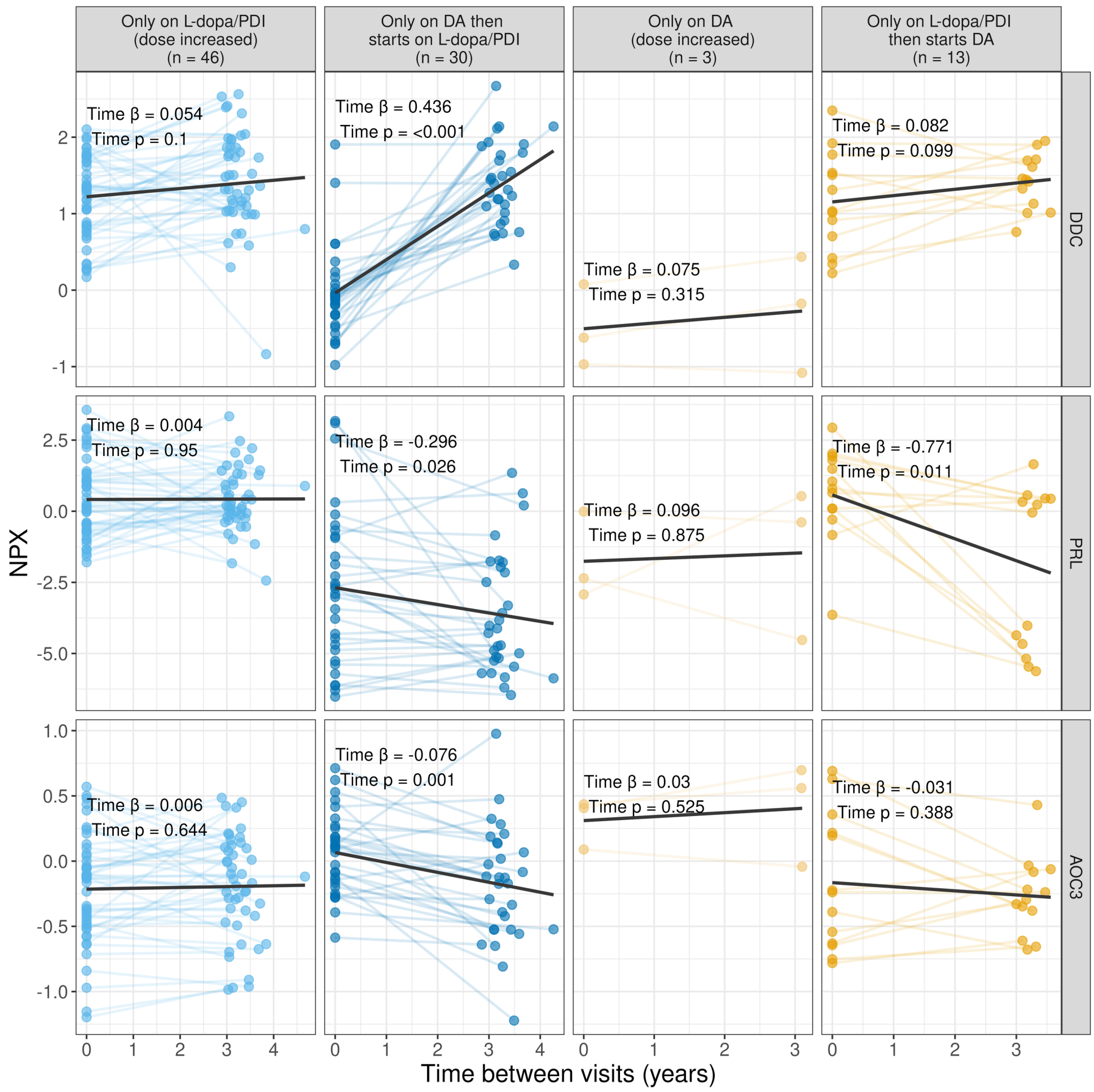

Raw serum protein NPX of samples plotted. Increase in LEDD defined as >100 between visits. Coefficient of time between visits and related p-value displayed. Linear mixed effects model fitted. Fixed effects: time, baseline age, sex, BMI and technical variables. Random intercept: participant ID. All p-values FDR-corrected.

Supplementary Table 3. LEDD and clinical variables

CSF DDC

Model	Coefficient (β)	p-value[†]
LEDD	0.049	0.221
UPDRS II	0.040	0.047
UPDRS III	0.006	0.393
H&Y	0.337	0.045
MoCA	-0.018	0.647

CSF PRL

Model	Coefficient (β)	p-value[†]
LEDD	-0.320	0.019
UPDRS II	-0.121	0.089
UPDRS III	-0.361	0.805
H&Y	-0.327	0.349
MoCA	-0.324	0.926

CSF AOC3

Model	Coefficient (β)	p-value[†]
LEDD	-0.004	0.779
UPDRS II	-0.008	0.230
UPDRS III	-0.001	0.142
H&Y	-0.005	0.216
MoCA	-0.003	0.536

Associations of proteins with LEDD and disease severity were analysed by simple linear-regression models. Covariates: age, sex, BM and technical variables. LEDD is shown scaled as LEDD/100 (mg/day). H&Y = Hoehn & Yahr stage
† nominal p-value

Sensitivity Analysis

Supplementary Figure 6. Linear associations between proteins and LEDD in serum at baseline

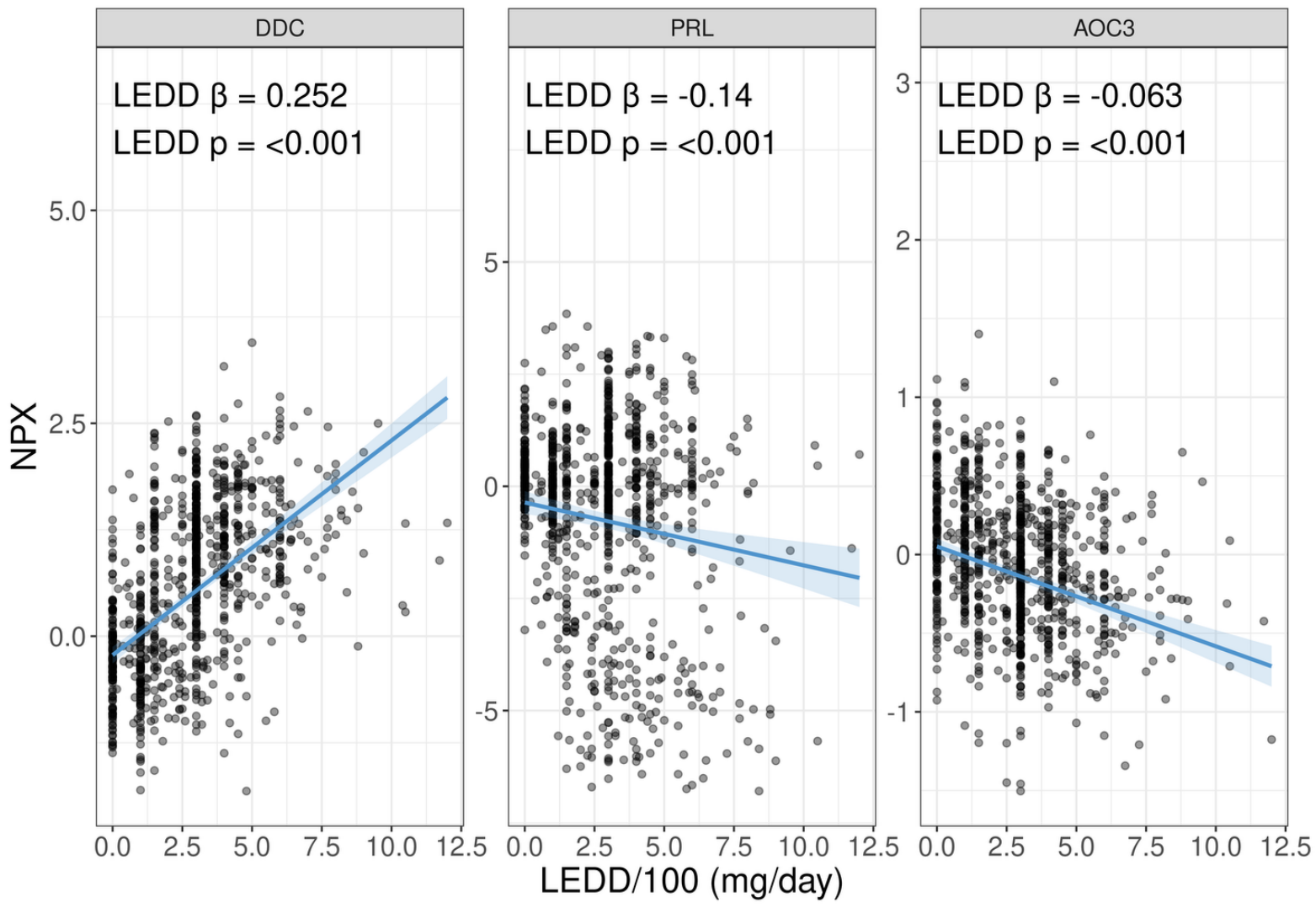

Raw serum protein NPX of all baseline PD samples (n = 894). Simple linear model fitted. Covariates: disease duration, age, sex, BMI and technical variables. All p-values FDR-corrected.

Supplementary Figure 7. Linear associations between LEDD and serum protein levels at baseline stratified by drug class

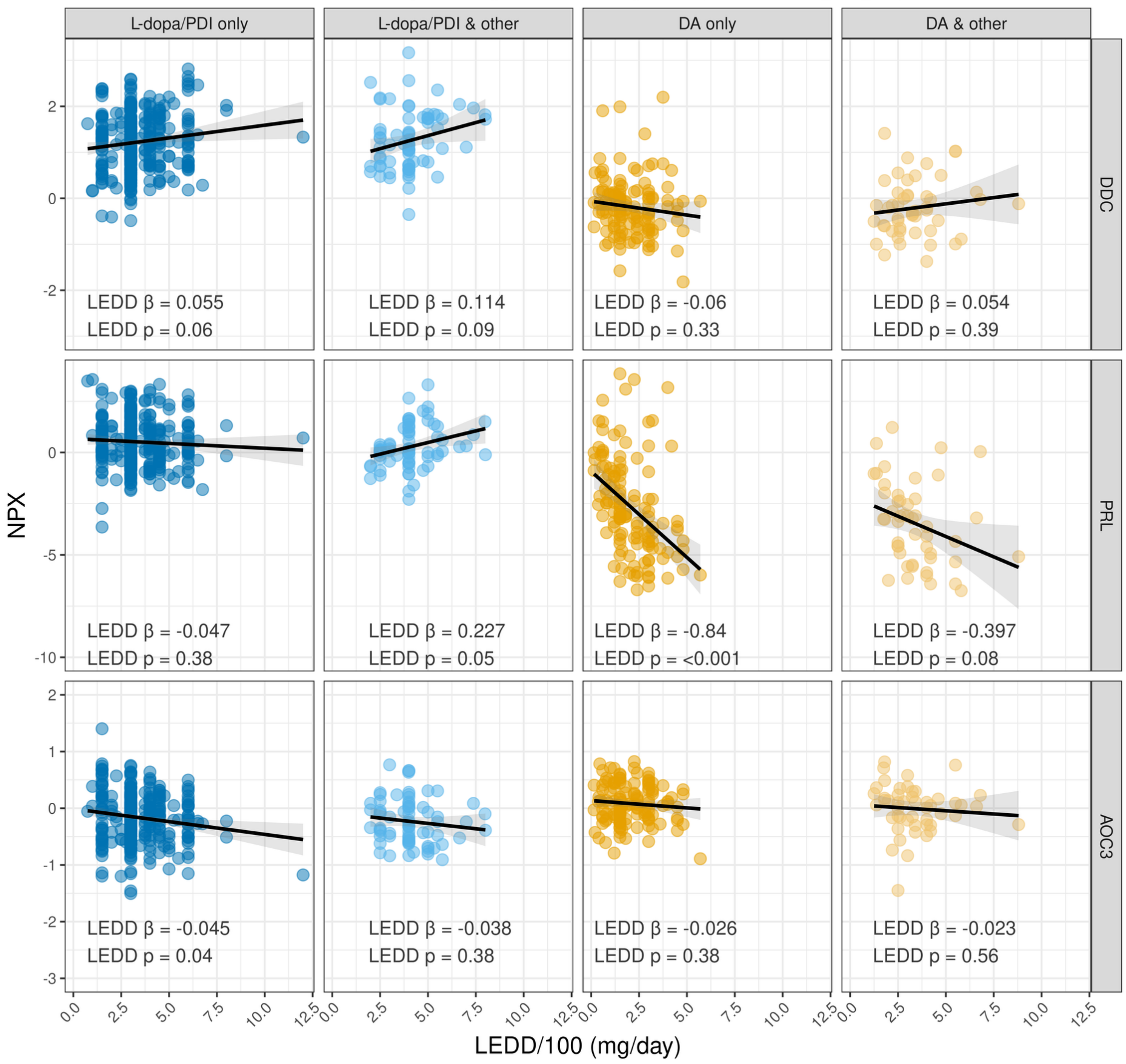

Raw serum protein NPX of samples plotted (n = 592). Simple linear model fitted. Covariates: disease duration, age, sex, BMI and technical variables. All p-values FDR-corrected.
L-dopa/PDI only = participants only on a combination drug of levodopa with peripheral decarboxylase inhibitor
L-dopa/PDI & other = participants on L-dopa/PDI and any other drug class, excluding dopamine agonists
DA only = participants only on a dopamine agonist
DA & other = participants on a dopamine agonist and any other drug class, excluding L-dopa/PDIs

Quality Control Summary

Supplementary Figure 8. Serum data: Quality control flow diagram

Total number of serum samples tested: **1912**

7 serum samples removed (no NPX generated – failed experiment)

- **New total: 1905**

16 samples identified as extreme outliers and removed

- **New total: 1889**

14 samples removed as they were not taken at visit 1.
Two samples removed due to insufficient metadata.
Two samples were removed as they represented a 2nd longitudinal sample years later

New total: 1873

Total number of assays tested: **5420**

2 assays (GBP1, MAP2K1) were tested in triplicate. The mean was calculated and carried forward to the downstream analysis

- **New total: 5416**

8 assays had an Olink WARN label (APOE, BLMH, CTSD, CTSV, ELANE, GAPDH, SBSM, SERPINB4). These were included in downstream analysis

Supplementary Figure 9. Extreme outlier detection (serum)

Outlier Detection

Outliers in the CSF proteomics dataset were identified using a combination of three complementary methods:

1. *Quality Control (QC) Plot Method:*

   Outliers were defined as samples exceeding 5 standard deviations from the mean interquartile range (IQR) or sample median across all proteins. This approach identified 0 extreme outliers (**A**).

2. *Principal Component Analysis (PCA) – Mahalanobis Distance:*

   PCA was performed on normalized protein expression (NPX) data, and Mahalanobis distance was used to identify statistical outliers with a confidence level of 99.999%, detecting 0 samples as outliers (**B**).

3. *PCA – Euclidean Distance from Centroid of Top Principal Components:*

   The top 10 principal components were used to calculate each sample's Euclidean distance from the centroid of the PD dataset. Zero samples exceeded 5 standard deviations from the mean distance.

A)

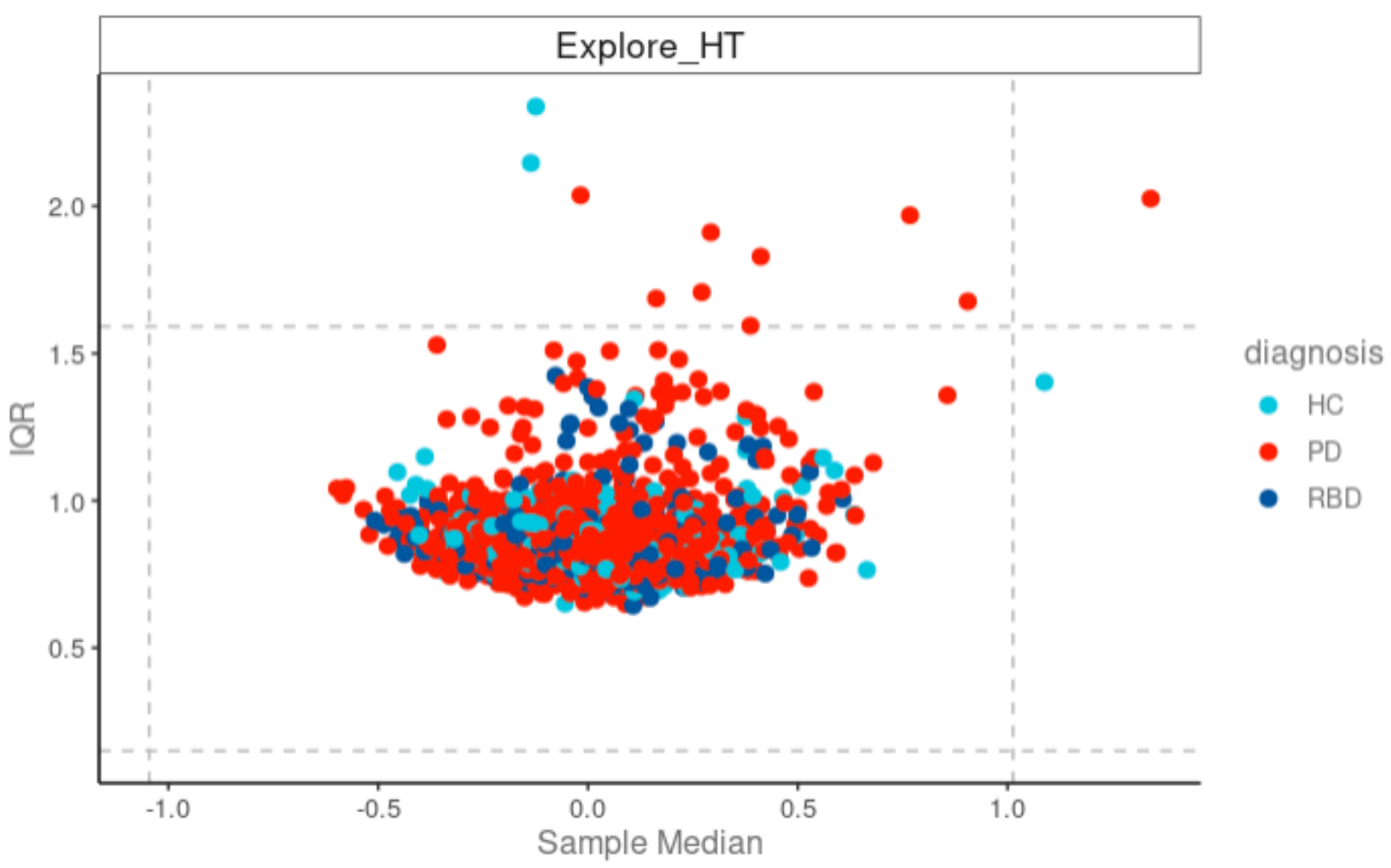

B)

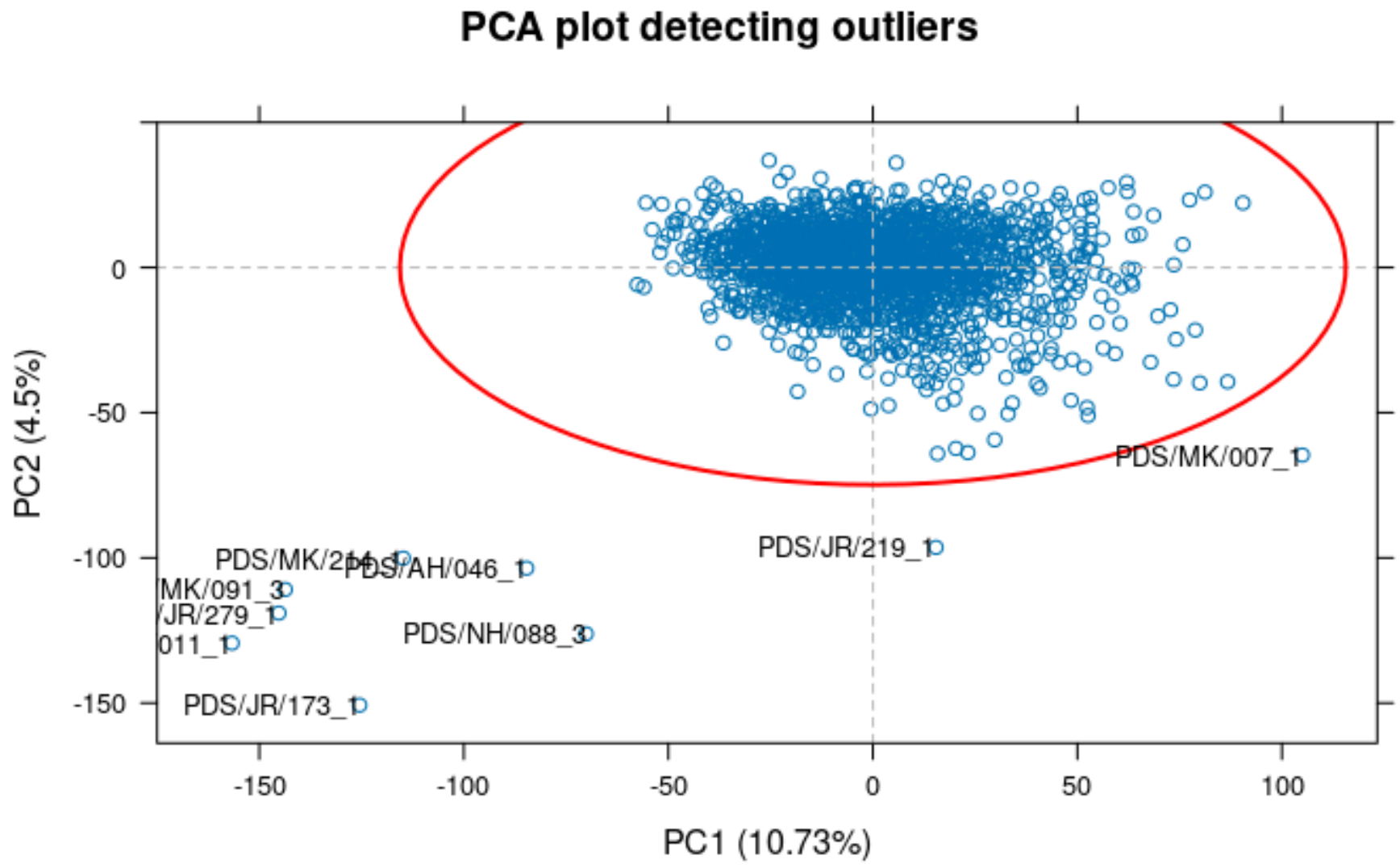

Supplementary Figure 10. CSF data: Quality control flow diagram

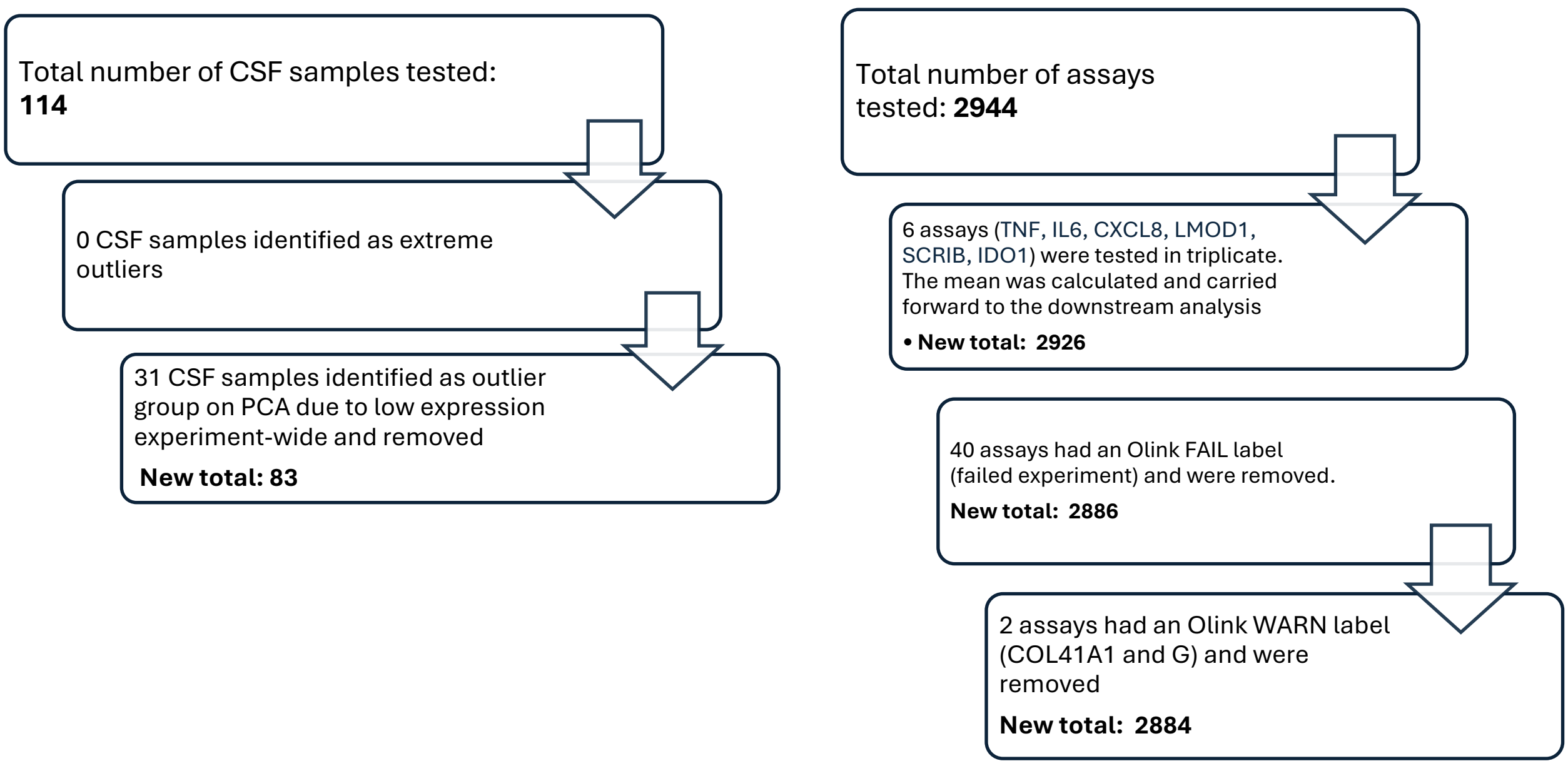

Supplementary Figure 11. Extreme outlier detection (CSF)

Outlier Detection

Outliers in the CSF proteomics dataset were identified using a combination of three complementary methods:

4. *Quality Control (QC) Plot Method:*

   Outliers were defined as samples exceeding 5 standard deviations from the mean interquartile range (IQR) or sample median across all proteins. This approach identified 0 extreme outliers (**C**).

5. *Principal Component Analysis (PCA) – Mahalanobis Distance:*

   PCA was performed on normalized protein expression (NPX) data, and Mahalanobis distance was used to identify statistical outliers with a confidence level of 99.999%, detecting 0 samples as outliers (**D**).

6. *PCA – Euclidean Distance from Centroid of Top Principal Components:*

   The top 10 principal components were used to calculate each sample's Euclidean distance from the centroid of the PD dataset. Zero samples exceeded 5 standard deviations from the mean distance.

C)

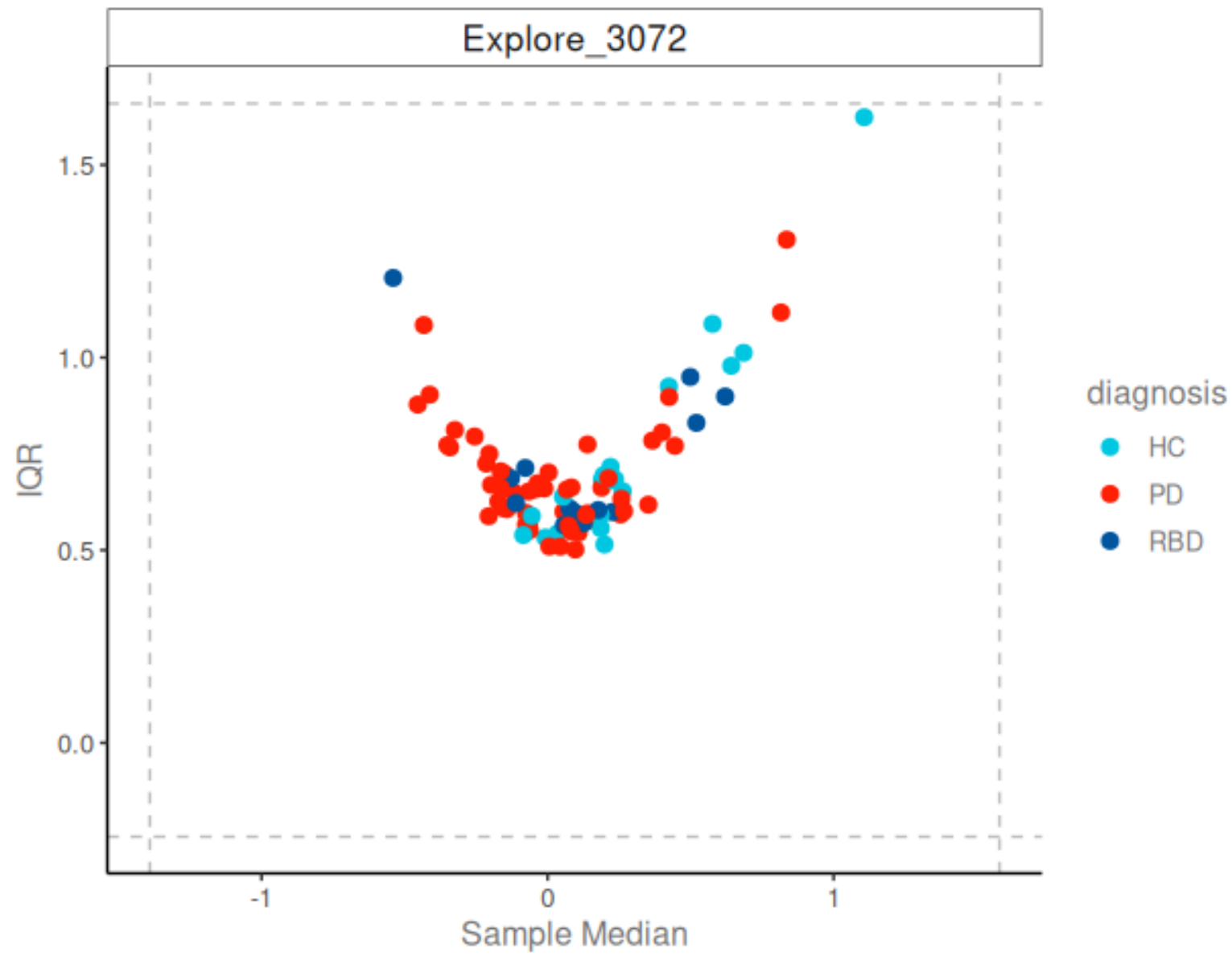

D)

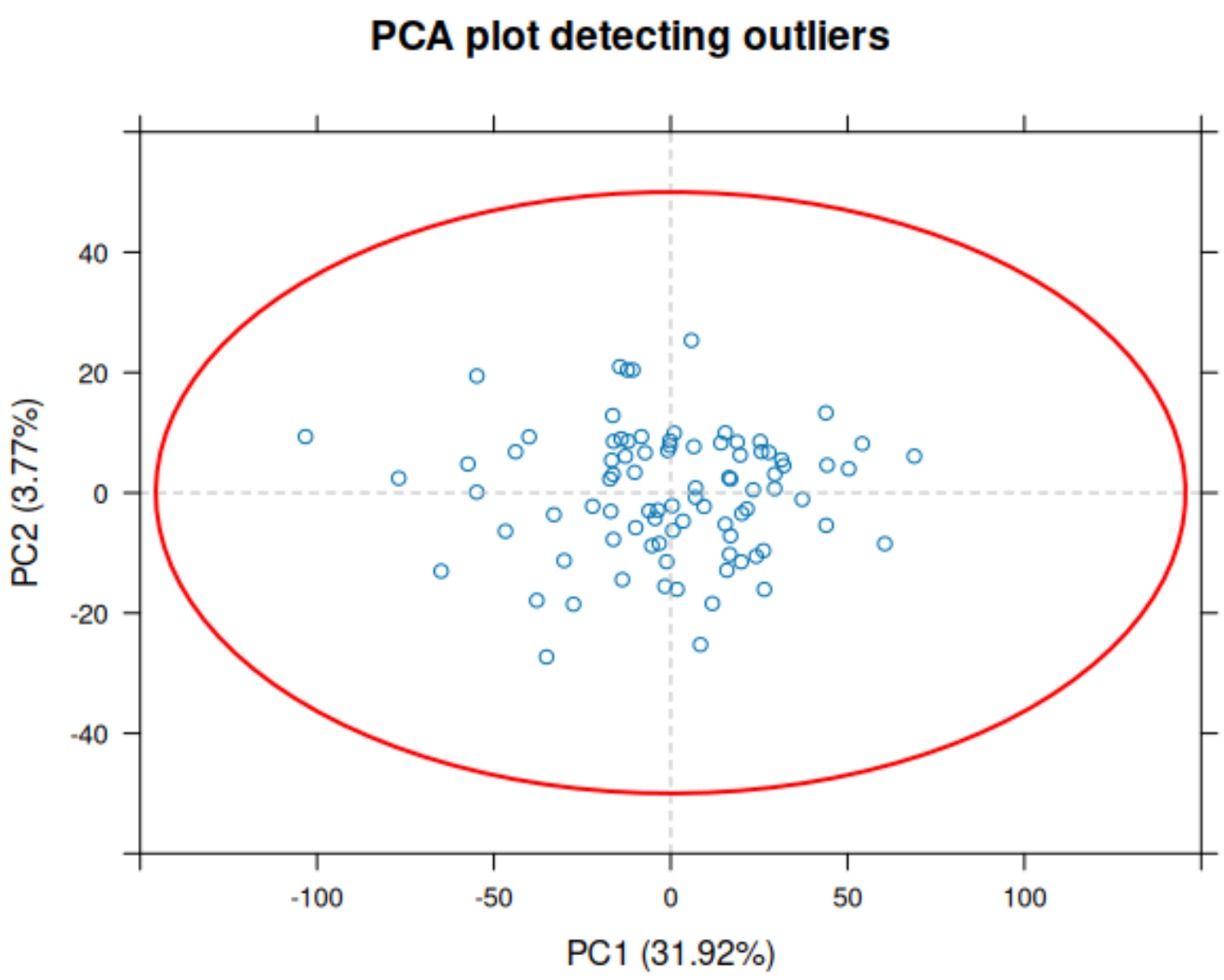

Supplementary Table 3. Interplate coefficient of variance (CV) for DDC, PRL and AOC3

Serum

Protein	CV
DDC	10.60%
PRL	11.62%
AOC3	7.76%

CSF

Protein	CV
DDC	7.76%
PRL	8.32%
AOC3	4.90%
